# Innate immunity and early liver inflammation

**DOI:** 10.3389/fimmu.2023.1175147

**Published:** 2023-05-02

**Authors:** Jordi Yang Zhou

**Affiliations:** ^1^ Department of Surgery, University Hospital Regensburg, Regensburg, Germany; ^2^ Leibniz Institute for Immunotherapy, Regensburg, Germany

**Keywords:** Inflammation, Innate immnuity, Hepatitis (general), NK cells, MAIT cell, Gd T cell, NKT (natural killer T) cell, kupffer cell (KC)

## Abstract

The innate system constitutes a first-line defence mechanism against pathogens. 80% of the blood supply entering the human liver arrives from the splanchnic circulation through the portal vein, so it is constantly exposed to immunologically active substances and pathogens from the gastrointestinal tract. Rapid neutralization of pathogens and toxins is an essential function of the liver, but so too is avoidance of harmful and unnecessary immune reactions. This delicate balance of reactivity and tolerance is orchestrated by a diverse repertoire of hepatic immune cells. In particular, the human liver is enriched in many innate immune cell subsets, including Kupffer cells (KCs), innate lymphoid cells (ILCs) like Natural Killer (NK) cells and ILC-like unconventional T cells – namely Natural Killer T cells (NKT), γδ T cells and Mucosal-associated Invariant T cells (MAIT). These cells reside in the liver in a memory-effector state, so they respond quickly to trigger appropriate responses. The contribution of aberrant innate immunity to inflammatory liver diseases is now being better understood. In particular, we are beginning to understand how specific innate immune subsets trigger chronic liver inflammation, which ultimately results in hepatic fibrosis. In this review, we consider the roles of specific innate immune cell subsets in early inflammation in human liver disease.

## Introduction

**Figure 1:**
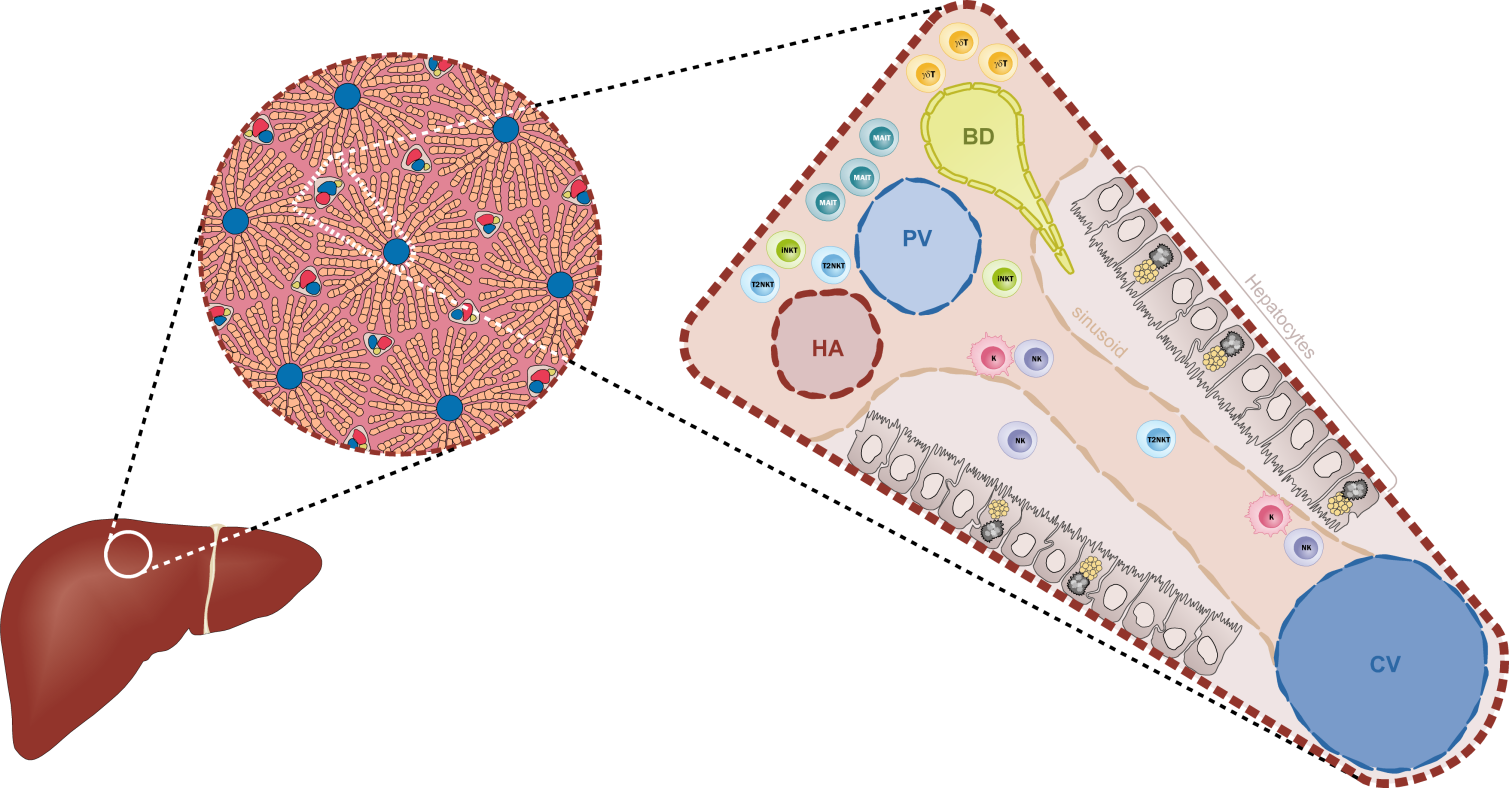
Diagrammatic representation of the liver architecture. The classical hexagonal lobule constitutes the anatomic unit of the liver. The lobule´s parenchyma is mainly formed by hepatocytes that are distributed along the sinusoids. The portal triad, formed by the hepatic artery (HA), the portal vein (PV) and the biliary duct (BD), carries the blood supply towards the centroid of the lobule where it is collected by the central vein (CV). Within the sinusoids, Kupffer cells (K) and Natural Killer cells (NK) are located in close proximity to the endothelium (beige). Other ILC-like cells such as iNKT cells and T2NKT cells are constantly surveying the sinusoids. Closer to the triad, especially near the BDs, there is a high frequency of MAIT cells and γδ T cells.

**Figure 2:**
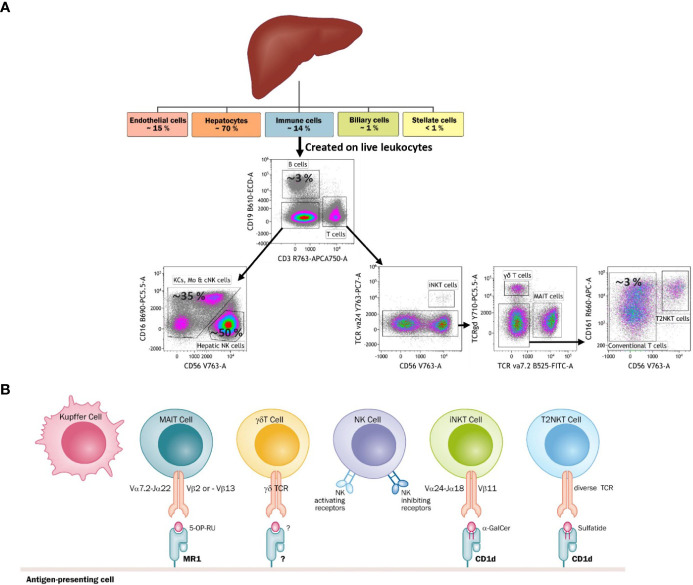
**(A)** Diagram tree of the approximate frequency of liver-resident cells and a FACS-based gating strategy to identify each cell type. The liver is mainly constituted by parenchyma (hepatocytes) and ILCs. Among ILCs, Kupffer cells and NK cells are the most abundant immune cells. The liver is also characteristic for having a niche of unconventional T cells, namely iNKT cells, T2NKT cells, γδ T cells and MAIT cells. **(B)** The main types of antigen recognition by unconventional T cells through their T-cell receptors (TCRs), Kupffer cells and NK cells. Kupffer cells and NK cells are activated through pattern recognition receptors. Additionally, NK cells have receptors that can sense healthy and stressed or dead cells.

Understanding the liver´s architecture and the niches formed by the different hepatic immune cells is equally important to deciphering their immune roles. The liver is subdivided into hepatic lobules, which consist of a portal triad (hepatic artery, portal vein and bile duct), hepatocytes arranged in linear cords between a capillary network (sinusoids) and a central vein (**Figure 1**). The blood flows from the portal triad to the central vein. The vascular system connecting the portal triad to the central vein is mainly constituted by liver sinusoidal endothelial cells (LSECs). Large fenestrae allow the exchange of macromolecules and components from the sinusoids with hepatocytes (1, 2). Interestingly, hepatocytes have different functions based on their zoning. Close to the portal triad, hepatocytes are the first to interact with gut-derived antigens whereas hepatocytes in proximity to the central vein are associated with detoxification (3). The gradual change in blood nutrients, oxygen and antigen load is correlated with significant changes in hepatocytes´ gene expression signature (3, 4). Immune cells could also perform different functions according to their position within the liver. The distribution of innate cells in the liver is based on different chemokines, adhesion molecules and surface receptors (5). KCs are located adherent in the sinusoids and emit extensions into the Disse space. KCs along with LSECs constitute part of the reticuloendothelial system, which clears debris and harmful compounds in the blood. 65% of intrahepatic lymphocytes consist of NK cells, NKT cells, MAIT cells and γδ T cells (6–8) (**Figures 2A, B**). NK cells are in close proximity to KCs in both mouse and human models, suggesting a physical co-dependence (9, 10). NKT cells are constantly surveilling the liver sinusoids and stop when they detect inflammatory signals (9). CXCR6 was identified as a receptor to regulate mouse intrahepatic NKT cell frequencies and its ligand CXCL16 is overexpressed in macrophages and endothelium near injury areas (10). Human γδ T cells were identified in portal sections and in association with biliary epithelium (11). Human MAIT cells are reported to reside predominantly around bile ducts (12). However, the distribution and frequency of innate cells during inflammation are drastically changed with the recruitment of immune cells to the site of inflammation (9).

## Kupffer cells

KCs are liver-resident macrophages that constitute 15% of the total human non-parenchymal liver cell count (13). They represent the primary barrier against pathogens and toxic compounds coming from portal circulation (14). KCs are antigen-presenting cells (APC) and play a crucial role in inducing liver tolerance through cell-to-cell contact, cytokines and other mechanisms such as dioxygenase-dependent sequestration of tryptophan (15). Under physiological conditions, KCs are the major reservoir of macrophages in the liver and can self-renew independently from the bone marrow (16). Upon activation, KCs secrete chemokine ligand 2 (CCL2) which promotes the infiltration of human circulating monocyte-derived macrophages. Increased frequency of CCR2^+^ monocytes participates in liver fibrosis in mouse models (17, 18) and is indicative of pathology in human acetaminophen-induced acute liver injury (19). However, it is not yet clear whether liver-resident and circulating macrophages are two distinguished populations with different functions. The majority of pathogens coming from portal circulation are trapped in the liver by KCs phagocytosis. KCs cooperate with other non-parenchymal liver cells to clear potential infections (20). KCs can also sense damage-associated molecular patterns (DAMPs) expressed in hepatocytes that induce the secretion of a variety of cytokines and chemokines to efficiently restore homeostasis (20). When liver diseases compromise KCs function, aggravation of the diseases can be foreseen due to secondary infections (21).

## Mucosal-associated invariant T cells

MAIT cells are an abundant subset of hepatic T lymphocytes. They constitute up to 30-40% of human hepatic CD8^+^ T cells (6, 7). Their roles in pathogen defense and tissue repair have been previously reported (22–24). MAIT cells have an invariant T cell receptor (TCR) that recognizes the nonpolymorphic class Ib major histocompatibility (MHC) class I-related protein (MR1) when loaded with antigens. MAIT cells recognize riboflavin derivatives which are necessary for metabolism of many bacteria. These cells are considered an evolutionary system to defend hosts from pathogens since mammals do not produce these metabolites. Under inflammatory conditions, hepatocytes present the riboflavin derivative 5-A-RU to MAIT cells and also secrete IL-7 which is known to shape MAIT cells towards a pro-inflammatory state (7, 25). Upon activation, MAIT cells secrete large amounts of pro-inflammatory and pro-fibrogenic cytokines such as IFN-γ, TNF-α and IL-17 (26). Studies in humans demonstrated that triggering MAIT cells in the absence of co-stimulation with cytokines induces wound repair and tissue regeneration (24). These studies suggest that under physiological conditions, MAIT cells probably contribute to tissue repair and regeneration since there is a constant influx of 5-A-RU present in human sera (27) but promote inflammation under acute inflammation. The high sensitivity for cytokines indicates that MAIT cells might be one of the first contributors to early inflammatory responses.

## Gamma-delta T cells

γδ T cells are non-conventional subset of T lymphocytes with a limited non-MHC-restricted TCR repertoire. They constitute around 1-10% of human circulating T cells (28). They can recognize a wide variety of antigens and can be activated *via* pathogen-associated molecular patterns (PAMPs), DAMPs or cytokines alone. Upon activation, cells can execute cytotoxic as well as effector functions. Moreover, γδ T cells also play a role in tissue homeostasis (29). In humans, the stratification of γδ T cells is based on the Vδ gene segments used to produce their TCR. Vδ1^+^ T cells are abundant in the epithelium (30) and protect tissues *via* recognition of non-classical MHCs such as CD1a, CD1c and CD1d (31). Vδ2^+^ T cells are the most abundant subtype in circulation and can clear infections in periphery organs (28, 32). They recognize phosphoantigens, which are non-peptide low molecular weight antigens. Vδ2^+^ T cells respond rapidly in a Th1-like fashion to high amounts of self-phosphoantigens (for example in tumor cells) or microbial phosphoantigens (33, 34). The butyrophilin 3A (BTN3A) family can trigger activation of Vδ2^+^ T cells upon stimulation with phosphoantigens (35). The heterodimer BTNL3/BTNL8 expressed in APC was reported to mediate the TCR-dependent activation of Vδ2^+^ T cells by binding of the intracellular domain of BTNL3 with phosphoantigens (36). Interestingly, the expression of *BTNL8* was not detectable in human PBMC but it was highly expressed in regulatory T cells after polyclonal stimulation (37). This suggests further investigation into the role of the butyrophilin family in the development of hepatitis and potential role in influencing Vδ2^+^ T cells. Vδ3^+^ T cells are a heterogeneous group of T lymphocytes enriched in the liver and also in some diseases such as leukaemia or chronic viral infection (38). They recognize antigens presented by CD1d molecules and respond by producing cytokines and killing of CD1d^+^ cells (38). Recent evidences suggest that γδ T cells may be involved in liver diseases as previously shown in other autoimmune diseases (28), especially due to the rapid and large secretion of IL-17 (39).

## Natural killer T cells

NKT cells are a rare subset of T lymphocytes comprising less than 1% of human peripheral blood T lymphocytes but enriched in the liver (8, 40). NKT cells are known to express NK cell markers like CD56, CD16 and CD161, and produce granzyme (40, 41). Their restricted TCR repertoire recognizes antigenic lipids presented by the MHC class I-like molecule CD1d (42, 43). Based on their TCR, NKT cells have been divided into two subsets. Type I NKT, or invariant (i)NKT cells, are the most studied group because they are enriched in mouse liver and have a semi-invariant TCR. The prototype ligand for iNKT cells is α-galactosylceramide (α-GalCer) (44). Type II NKT cells (T2NKT) consist of a subset with more diverse TCR. The major ligand recognized by T2NKT cells is sulfatide, which is a glycolipid enriched in the myelin of the central nervous system, pancreas, kidney and liver (45). It is difficult to study T2NKT cells because there is a lack of tools to identify and characterize them. Recently, we proposed a novel strategy to isolate and characterize T2NKT cells in humans but the low number of cells in blood is still a limitation (40). The role of iNKT cells and T2NKT cells in liver diseases have been mainly studied using transgenic mice models of CD1d-knockouts or TCRVα14-knockouts, which lack iNKT cells. These studies suggest that, in general, iNKT cells play a pro-inflammatory phenotype whereas T2NKT cells suppress inflammation through direct and indirect inhibition of inflammatory cells, including iNKT cells (46–49). We described a novel subpopulation of T2NKT cells that expresses regulatory T cell markers such as FoxP3 and CD25 (40). FoxP3^+^ T2NKT cells were found both in the periphery and in the liver and may explain some of the regulatory functions reported previously.

## Natural killer cells

NK cells are a major component of the liver’s innate immune cell compartment. They account for almost 50% of human intrahepatic lymphocytes (50). Human hepatic NK cells are classified into three different subsets based upon their transcriptional, phenotypical and functional features (50). Liver-resident NK cells are CD56^bright^ CD69^+^ CXCR6^+^ CCR5^+^ and highly cytotoxic (51–54). These cells are long-lived tissue-resident subsets (55). Interestingly, a subset of liver-resident CXCR6^+^ NK cells was described as having a memory-like responsiveness against - vesicular stomatitis virus (VSV), human immunodeficiency virus (HIV) and influenza (56). Memory-like NK cells produce higher amounts of IFN-γ after rechallenge with the virus. The third NK cell subset is transient circulating NK cells, which are CD56^dim^ CD69^-^ CXCR6^-^ CCR5^-^ and show less cytotoxic activity. They can secrete high amounts of pro-inflammatory cytokines such as TNF-α and GM-CSF (57–59). The regulation of NK cell activity consist on a balance between activating and inhibitory receptors displayed on their surface (60). NK cells survey the liver and induce apoptosis in infected or aberrant cells *via* different mechanisms such as FasL or TRAIL (61, 62). Under inflammatory conditions, NK cells kill hepatic stellate cells (HSCs) to resolve inflammation and limit liver fibrosis *via* granzyme-induced apoptosis and IFN-γ secretion (62, 63). NK cells are fundamental for the proper protection of the liver and aberrant functions have been reported in several liver diseases. Over the past decade, studies on NK cells suggest very heterogeneous populations with distinctive transcriptomes and cellular interactions (64).

## Innate immune cell subsets and early liver inflammation

**Figure 3:**
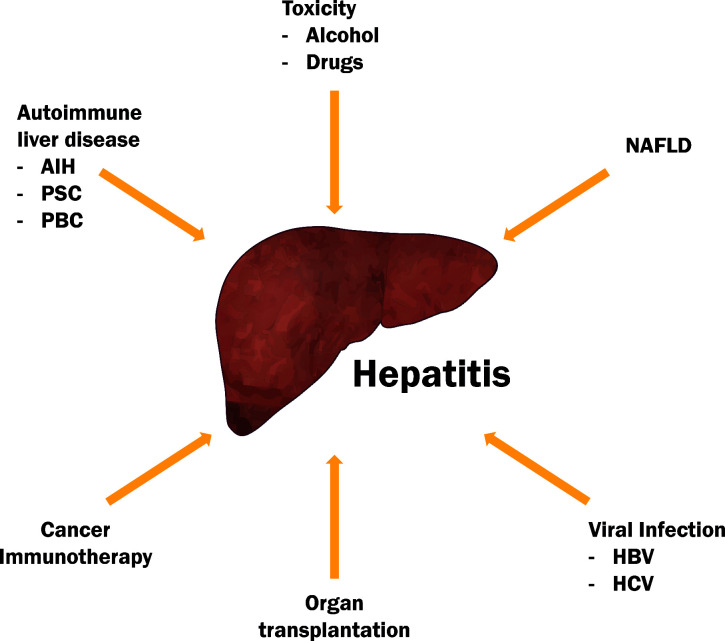
Hepatitis is a hallmark of the majority of liver diseases.

Liver inflammation is the first step to resolving and healing from different hepatocellular stress. When not effective, inflammation can become pathogenic. Hepatitis is a hallmark of liver disease (65) (**Figure 3**). It is important to identify which cells are precursors of early liver inflammation to avoid unnecessary harm. A recent report highlights the importance of the inflammasome in early inflammation (66). KCs express a variety of pathogen recognition receptors (PRRs) to cover a wide range of dangers. Some of these dangers overactivate the inflammasome, which triggers pyroptosis, a form of cell death accompanied by cell membrane rupture and release of pro-inflammatory IL-1β and IL-18 (67). These cytokines are responsible for the recruitment and activation of innate immune cells (68, 69). The direct cytotoxic and effector functions of innate immune cells can restore homeostasis. However, innate immune cells can also have early involvement in disease processes when the danger is not resolved (e.g. chronic viral infection) or because of repeated insults (e.g. alcohol or drug abuse) (**Supplementary Table 1**) . Innate immune cells can also recruit other immune cells from the liver and peripheral circulation. Overall, innate immune cells are suggested to be the precursors of the inflammatory niche because of their optimal location, preactivated state, enrichment in the liver and strong effector functions.

## Viral hepatitis

Hepatotropic viruses such as hepatitis A, B, C, D and E (HAV, HBV, HCV, HDV and HEV) possess mechanisms to escape from the hosts´ antiviral immunity. When the viruses replicate, often the innate immunity detects viral components, hence triggering an acute inflammatory response resulting in the killing of infected hepatocytes. Since the infection is not properly resolved, viruses remain in a latent state and replicate opportunistically. This progressively leads to chronic liver inflammation (70). In particular, HBV and HCV are the main causes of chronic liver disease and are estimated to affect 257 million (data from WHO 2015) and 115 million people (71), respectively. Together they represent the most common cause of liver cirrhosis, liver cancer and viral hepatitis-related deaths (72).

HBV is directly mutagenic and induces low-grade inflammation progressing into HCC (73). HBV-infected hepatocytes release PAMPs such as glycoproteins, secreted HBsAg or free viral nucleic acids that are recognized by the innate immune system. Human KCs release pro-inflammatory cytokines to orchestrate an antiviral response which also arrests hepatocyte replication, hence viral replication (74). Studies in mice demonstrated the antiviral roles of NK cells and NKT cells (75, 76). HBV patients present higher levels of NK cells in blood compared to HBV-negative controls (77, 78) and are deemed as the major contributors to HBV clearance (79). A positive correlation was found between NK cell activation levels and HBV clearance (79). NK and NKT cell numbers from peripheral blood correlated to the frequency of HBcAg-specific cytotoxic T lymphocytes (CTLs) (80). However, infiltration of circulating NK cells can contribute to liver injury (81). NK cells from HBV patients produced higher levels of TNF-α and induced *in vitro* expression of TRAIL in hepatocytes (82). This study showed that infiltrated circulating NK cells could induce apoptosis of non-infected hepatocytes *via* TRAIL (82). Additional studies in mice and patients show that NK cells could also exacerbate liver injury *via* TNF-α, Fas/FasL and NKG2D/NKG2DL pathways (83, 84). NKT cells and KCs secrete induced nitric oxide synthase (iNOS) as a viral eradication mechanism (85, 86). Moreover, the frequency of NKT cells was increased to normal values with virus clearance (80). These results suggest that circulating NK cells and NKT cells are recruited in the liver causing a reduction in their frequencies in blood. In contrast, peripheral MAIT cells were significantly decreased in HBV-related liver failure patients compared with chronic HBV patients (87). The study suggested that MAIT cells are recruited in the liver and promote a strong inflammatory response damaging the liver. MAIT cells were also reduced in patients with middle/late-stage compared with early-stage liver failure (87). Similar to NK cells and NKT cells, patients that showed disease improvement had an increment in the frequency of MAIT cells (87). In another two studies exploring changes in peripheral γδ T cells in HBV patients, γδ T cells were less abundant in liver failure patients and correlated with disease severity (88). Activation of γδ T cells with PMA/Ionomycin induced the greatest amount of pro-inflammatory TNF-α and IL-17 in liver failure patients (89). However, another study indicated that γδ2 T cells exhibited impaired proliferation and chemotaxis (90). The same study showed *in vitro* that γδ2 T cells inhibit Th17 T cells through cell-to-cell contact and produce high amounts of IFN-γ (90). These results suggest that NK cells and NKT cells are the first-line of defense against HBV infection. Failing to clear the infection, MAIT cells and γδ T cells contribute to chronic inflammation. IFN-α therapy is effective in 20-30% of chronic HBV patients (91). The low response rates may be attributed to the wide spectrum of different clinical conditions. Based on the current understanding of the role of NK cells in HBV clearance, IFN-α is likely to improve the cytotoxic function of liver-resident NK cells by targeting HSC cells and reduce fibrosis (92). It is necessary to investigate whether IFN-α therapy response is subjected to the frequency of circulating NK cell infiltration.

HCV-induced inflammation is partly triggered by non-structural proteins of the virus (93) but the major contributor to HCV-hepatitis are the inflammatory immune cells. *In vitro* studies show that HCV-infected hepatocytes produce several pro-inflammatory cytokines including IL-6, IL-8, MIP-1α and MIP-1β as a response to IL-1β secreted by HSCs (94) or IL-1β and TNF-α by KCs (95). Similar to HBV infection, human circulating MAIT cells were generally reported to be depleted with markers of exhaustion and hyperactivation (96–98). Additional studies suggest that hepatic MAIT cells are major contributors to hepatitis and fibrosis given the nature of the cells. Repetitive IL-12 stimulation or IL-7 secretion by hepatocytes was a sufficient stimulus to induce secretion of the pro-inflammatory cytokines IFN-γ, TNF-α and IL-17 (7, 26). Intrahepatic γδ T cells were shown to be cytotoxic against human hepatocytes in culture (99). We have recently identified a subset of CD8^+^ γδ T cells that were more abundant in baseline peripheral blood of melanoma patients that had hepatitis after ICI therapy versus non-hepatitis cohort. ICI therapy might induce γδ T cells cytotoxic activity against hepatocytes as observed in HCV infection. NK cells were shown to be compromised in HCV patients allowing the virus to replicate (78, 100). IFN-α therapy induced activation of NK cells and further improved the clearance of the virus (101). NKT cells were also reported to play a role in HCV resolution and progression. The frequency of activated CD38^+^ or CD69^+^ iNKT cells strongly correlated with alanine transaminase levels (102). Increased levels of activated iNKT cells were observed during acute inflammation and chronic HCV infection without apparent functional differences (102). The frequency of activated iNKT cells declined spontaneously in resolving patients (102). These data suggest that HCV infection could be mainly managed by NK and NKT cells. Viral clearance also involves other ILC-like cells such as MAIT cells and γδ T cells. Under inflammatory conditions, host hepatocytes switch to an antiviral state to prevent further viral replication. If the infection is not properly resolved, we propose a model where NK cells and MAIT cells have an exhausted phenotype while iNKT cells and γδ T cells promote pathogenesis by targeting infected hepatocytes.

## Alcohol-induced hepatitis and drugs

The liver is vital for the detoxification of substances that are harmful to the body. Liver detoxification consists mainly of converting ingested drugs into water-soluble metabolites *via* xenobiotic biotransforming enzymes (103). This allows drugs to be efficiently secreted through urine. However, in an attempt to solubilize drugs, some compounds are converted into their active form. Acetaminophen, also known as paracetamol, leads to reactive metabolites causing apoptosis and necrosis of hepatocytes (104). In the case of alcohol, free radicals and acetaldehyde are harmful by-products that can lead to significant liver damage over time. Drugs and alcohol can also damage the intestine barrier leading to more bacteria translocation to the bloodstream (105, 106). The influx of gut microbiota and its metabolites activate the immune system through PAMPs and DAMPs (107–110). KCs were reported to be major contributors to the development of alcohol-related liver disease (ALD). Intestine permeability is directly associated with KC activation (111, 112). Exposing mice to LPS and alcohol-derived reactive oxygen species (ROS) has shown to induce TNF-α secretion by KCs (113, 114). In a paracrine manner, IL-1ß secretion by KCs had a significant effect on the pathological progression of ALD (115). A rat model of ALD with depletion of KCs resulted in impaired progression of the pathology suggesting a key role of KCs (116). NK cells were less frequent in alcoholic patients (117) and were less cytotoxic compared to healthy individuals (118). A reduced expression of the activating receptor NKG2D and production of IFN-γ in mice suggests that NK cells cannot efficiently kill activated HSCs (119). Chronic ethanol feeding in mice increased CD1d by enterocytes (120). Similarly, patients affected by alcohol misuse also show increased expression of CD1d in the small intestine (120). An *in vitro* study showed that CD1d increased the loading of αGalCer following increasing concentrations of ethanol and thus, could increase stimulation of iNKT cells (121). Many studies in mice suggest that iNKT cells have a pathogenic role in the development of ALD. It was reported that iNKT cells crosstalk with KCs through IL-1β, promote inflammation and recruit neutrophils (122, 123). CD1d blocking antibodies could partially prevent liver injury (123). Intestinal iNKT cells were observed to migrate to the liver and, collectively with liver iNKT cells, showed a chronic activated phenotype with downregulation of TCR, increased apoptosis and FasL expression (120). *In vitro* experiments from the same study confirmed that iNKT cells could kill hepatocytes *via* Fas-FasL mechanism (120). Activation of T2NKT cells by sulfatide inhibited iNKT cell hepatic damage (124, 125). In a concanavalin A-induced hepatitis mouse model, injection of lysophosphatidylcholine (LPC) activated T2NKT cells and prevented liver injury by iNKT cells (125). Another study described the crosstalk of T2NKT cells with plasmacytoid dendritic cells and recruitment of anergic iNKT cells to the mouse liver *via* IL-12 and MIP-2 (126). As mentioned above, our group recently identified a novel population of human FoxP3^+^ T2NKT cells that might exert immunoregulatory functions in this scenario (40). Alcoholic-related cirrhosis and severe alcoholic hepatitis patients had a dramatic depletion and hyperactivated circulating MAIT cells (127, 128). Dysfunctional MAIT cells could explain the susceptibility to infection of these patients (127, 128). In another study, MAIT cells had an exhausted phenotype and partially recovered with patient´s alcohol abstinence (129). MAIT cells may contribute to the pathogenesis of ALD *via* IL-17 secretion (129). Surprisingly, only a few reports have described the role of γδ T cells in ALD. In a mouse study following binge ethanol drinking, γδ T cells were described to produce higher amounts of IL-17A than non-binge ethanol-drinking mice (130). The activation of γδ T cells was IL-1ß-dependent, possibly by KCs (130). However, under acute-on-chronic ethanol consumption, γδ T cells did not produce further IL-17A. Instead, CD4+ T cells were the major contributors. This suggests that KCs could play a predominant role in the development of ALD. KCs orchestrate an inflammatory response that involves pro-inflammatory iNKT cells and γδ T cells. Alcohol could directly affect MAIT cells and NK cells causing depletion and impaired functions such as the inactivation of HSCs by NK cells, and tissue repair by MAIT cells.

## Non-alcoholic fatty liver disease

Non-alcoholic fatty liver disease (NAFLD), characterized by an excessive accumulation of fat in hepatocytes, is the most common indication for liver transplant in Western countries and the leading cause of liver transplantation in women (131, 132). It is estimated that 23-25% of the global population have NAFLD to some degree (133). Etiologically, it is suggested that the adipose tissue from patients with NAFLD predisposition release free fatty acids (FFA) and pro-inflammatory mediators into the circulation (134, 135). As a result, an inflammatory response is triggered in the liver. Lipotoxicity, mitochondrial dysfunction and endoplasmic reticulum stress are key inducers of the inflammatory cascade (136). Higher frequencies of KCs were observed in liver biopsies of non-alcoholic steatohepatitis (NASH) patients (137). Depletion of KCs in rats exposed to a high-fat diet (HFD) prevented the development of steatosis (138). *In vitro* experiments showed that TNF-α was responsible for the increased accumulation and the reduced oxidation of fatty acids in hepatocytes (139). Immunohistological stainings revealed a complex crown-like structure consisting of KCs surrounding dying steatotic hepatocytes. Cholesterol crystals are accumulated in the center of these structures (140). Interestingly, previous exposure of KCs to cholesterol crystals showed to precondition the cells towards a pro-inflammatory innate memory-like state (141). Similar observations were taken from macrophages cultured with oxidized low-density lipoproteins (142). Likewise to the effect of alcohol, NK cells of obese individuals had lower NKG2D expression (143) and impaired cytotoxicity (144, 145). Another study showed that there were no differences between NK cells from healthy individuals and NAFLD, while higher expression of NKG2D in NK cells was found in NASH patients (146). Data from mice and humans suggest that iNKT cells have a dual role in NAFLD. More specifically, it is hypothesized that iNKT cells have a protective role during early stages of simple steatosis. In different mouse models of hepatosteatosis, like ob/ob mice, animals fed with HFD or a choline-deficient diet, iNKT cells were apoptotic and showed decreased intrahepatic frequency (147–149). Adoptive transfer of hepatic mononuclear cells but not CD1d^-/-^ mononuclear cells regulated hepatic steatosis *via* IL-10 (150). However, in other instances, opposite results were reported. Mice fed with HFD developed adipose tissue inflammation and glucose intolerance (151). This was significantly exacerbated by αGalCer-dependent activation of iNKT cells (151). In the liver, iNKT cells could be directly activated *via* hepatic CD1d molecules, exacerbate steatosis and decrease insulin sensitivity by promoting a pro-inflammatory cytokine environment (152). This could suggest that iNKT cells play a protective role during early stages of simple steatosis but exacerbate the disease in chronic steatosis. It would also be interesting to study the potential effect of iNKT cell migration from tissues like the intestines as discussed earlier. T2NKT cells might also play dual roles. In HFD mice, T2NKT cells initiate inflammation in the liver and adipose tissue and promote obesity and insulin resistance (153). However, adoptive transfer of T2NKT cells in HFD obese mice induced prolonged weight loss and glucose tolerance (154). The heterogeneity and impact of fat in intrahepatic T2NKT cell populations remains unclear. The frequency of human NKT cells is decreased in steatosis (155) but increased accordingly to the progression of NAFLD, especially IFN-γ^+^ and IL-4^+^ cells (156–158). NASH patients had a 4-5 fold relative increase in liver NKT cells (158). CD1d expression was reported to be increased in liver immunohistochemical samples of NAFLD and correlated with disease progression (156). Taken together, NKT cells are reduced in the early stages of simple steatosis. A pro-inflammatory response is protective against obesity. In advanced NAFLD, NKT cells are increased and pathogenic. Circulating MAIT cell frequency was reported to decrease while the number of intrahepatic MAIT cells was increased in NAFLD patients’ livers and it tended to be greater with disease progression (159). MAIT cells from NAFLD patients had increased secretion of IL-4 and reduced expression of IFN-γ and TNF-α (159). The current knowledge about the role of γδ T cells in NAFLD is mostly based on mice models. γδ T cells can recognize molecules presented by CD1d and its differentiation is dependent on hepatocyte CD1d (160). γδ T cells are high producers of IL-17A in steatohepatitis (161), a key cytokine known to induce fibrosis and ROS production (162, 163). In HFD mice, IL-17^+^ γδ T cells are elevated (164). Additionally, adoptive transfer and gene knockout experiments in HFD mice demonstrated that γδ T cells exacerbate steatohepatitis and liver damage (160, 161). In humans, NAFLD patients showed decreased frequencies of Vδ2^+^ T cells, but elevated frequencies of Vδ2^-^ T cells compared to healthy controls (143). Overall, the progression of NAFLD to NASH is a process derived from the increased cellular oxidative stress that leads to the activation of inflammatory pathways (165). Accumulation of ROS induces the expression of TNF-α which can trigger necrotic cell death (166). In line with these results, NK cells were suppressed by ROS (167). KCs develop an apparent pro-inflammatory immune memory state by contact with cholesterol crystals. γδ T cells promote pathogenesis through IL-17 secretion, while NKT cells and MAIT cells exacerbate steatosis by secretion of Th2 cytokines which also contributes to fibrosis (168).

## Liver autoimmunity

The three main autoimmune liver diseases are autoimmune hepatitis (AIH), primary biliary cirrhosis (PBC) and primary sclerosing cholangitis (PSC). AIH affects portal tracts and liver lobules by lymphoplasmacytic infiltrates while PSC and PBC mainly affect bile ducts. The etiologies of these diseases are yet unknown, but several studies suggest a common immune-mediated liver injury. The dysregulation of immune regulatory networks causes the activation and expansion of autoreactive T cells and B cells (169, 170). The innate system plays an important role in the regulation of the adaptive system. In AIH, an increased frequency of cytotoxic circulating NK cells in the liver was observed in an experimental mouse model of AIH (171). In humans, the frequency of circulating CD56^bright^ NK cells was higher in untreated AIH, while the frequency of circulating CD56^dim^ NK cells was reported to be reduced in active AIH patients or while in remission (171, 172). Our knowledge about NKT cells in liver autoimmunity is mainly based on mouse models. In AIH, concanavalin-induced hepatitis is the preferred model. iNKT cells were reported to upregulate FasL expression to mediate cytotoxicity against hepatocytes (173). Activation of iNKT cells *via* α-GalCer exacerbates the disease and is suggested to be carried out *via* IL-4 and TNF-α secretion (174, 175). Inflammation was also promoted *via* the secretion of IL-17 (176). MAIT cells were reported to be depleted and exhausted in the periphery in patients (177). Chronic stimulation of MAIT cells due to an increased influx of bacteria antigens and chronic inflammation may lead to MAIT cell function impairment. Induction of the exhausted state by repetitive stimulation with IL-12 and IL-18 showed that MAIT cells reduced IFN-γ production but maintained expression of the proinflammatory cytokine IL-17 (177). The frequency of circulating γδ T cells was increased in patients with AIH, PSC and PBC (8). Vδ1^+^ T cells, known to produce high levels of IFN-γ and granzyme B, were especially incremented in patients with AIH (178). Another study showed that γδ T cells with low expression of TOX were enriched in AIH patients and had prediction potential (179). TOX deficiency was suggested to promote the expression of IL-17A in γδ T cells (179). In general, IL-17 secretion was reported in iNKT cells, MAIT cells and γδ T cells. Although the clinical profile of the distinctive autoimmune liver diseases is different, current studies support common immunological pathways. Taking for instance the role of circulating NK cells, the frequency of these cells was reported to be increased and a higher expression of cytotoxic molecules such as perforin was found in PBC and PSC patients compared to healthy individuals (180, 181).

## Liver transplantation

Liver transplantation represents a major hepatic injury. One of the unavoidable injuries is caused by oxygen deprivation. After liver resection, blood flow is restricted for a period of time and the organ becomes hypoxic. This leads to different forms of cell death like apoptosis, ferroptosis, pyroptosis and necrosis (182). After reperfusion, innate immune cells from the recipient migrate to the liver and induce inflammation or tolerance (183). The degree of ischemia-reperfusion injury (I/R) is correlated to the risk of liver rejection (184, 185). I/R injury increased the expression of monocyte chemoattractant protein-1 (MCP-1) and it was associated with poorer graft function (186). This observation was correlated with the increased recruitment of monocytes 2 hours after reperfusion (186). The role of NK cells is dependent on activating and inhibitory receptors expressed in hepatocytes as well as cytokines secreted by neighbour cells. In I/R injury, components of the inflammasome in KCs like NLRP3 and AIM2 are hyper-activated (187, 188). Inflammasome-derived IL-18 secretion can induce FasL (189) and IFN-γ production in NK cells (190). IFN-γ was reported to induce expression of Fas receptor in hepatocytes and neutralization of IFN-γ secretion by NK cells could protect mice from tissue damage (191). Due to the increased demand for livers and the increasing prevalence of NAFLD, the debate of using steatotic livers for transplant is on the table (192). Steatosis is deemed to cause oxidative stress in the liver, which worsens the graft´s condition with I/R injury. In a retrospective, exploratory study, steatotic livers showing signs of I/R had a significantly worse one-year survival rate, while the survival rate was not conditioned in healthy livers´ by I/R injury (193). In this study, γδ T cells were suggested to exacerbate liver rejection in steatotic livers (193). NKT cells were reported to promote I/R injury. After reperfusion, NKT cells rapidly expand in the liver and produce IFN-γ (194, 195). Depletion of NKT cells with antibodies or both NKT cells and NK cells significantly reduced I/R injury (196). The role of MAIT cells in liver I/R injury remains to be elucidated. In focal cerebral ischemia, MAIT cells were reported to play a pro-inflammatory role (197).

## Immunotherapy-associated liver reactions

Cancer immunotherapies, especially immune checkpoint inhibitor (ICI) therapy, have opened new clinical perspectives for cancer patients and is fast becoming one of the main pillars of cancer treatment. ICI therapy uses monoclonal antibodies blocking T cell receptors that are used by cancer cells to evade the immune system. Immune-related adverse events (irAEs) are the result of immune activation derived from ICI therapy. The incidence of ICI-derived hepatitis is approximately 1-3% for programmed cell death 1 (PD1) inhibitors and 3-9% in cytotoxic T-lymphocyte-associated protein 4 (CTLA4) inhibitors (198). The combination of α-PD1/CTLA4 increases the rate of hepatitis (198). CTLA4 plays an important role in downregulating the immune response. The expression of CTLA4 is upregulated in T cells after activation and competes with the costimulatory receptor CD28 to bind to its ligand CD80/CD86 on APC (199). PD-1 is expressed on T cells and B cells and it promotes self-tolerance. Upon binding to its ligand PD-L1, it drives T cell apoptosis or regulatory phenotype. Thus, ICI therapy can arguably impair liver immunotolerance. In acute liver injury, α-PD1 therapy improved the bacterial clearance function of KCs (200). A study treating melanoma patients with α-PD1 showed that NK cell frequency in blood was not affected while NKT frequency was significantly increased (201). Another study observed no changes in either the number or function of MAIT cells in melanoma patients treated with α-PD1 therapy (202). γδ T cells showed no apparent functional changes upon PD-1 blockade *in vitro* (203). The frequency of γδ T cells in melanoma patients treated with a combination of α-PD1/CTLA4 remained unchanged (204). Overall, these data suggest that innate immune cells are not drastically affected by ICI therapies, with the exception of KCs and NKT cells. Immune-suppressive KCs expresses PD-1 to suppress T lymphocytes in acute liver injury (200). α-PD-1 therapy has shown to invigorate bacteria clearance, but it also suggests that KCs may have impaired tolerogenic function to self-antigens reactive T cells. NKT cells also responded to α-PD-1 therapy and exert increased anti-tumor functions by secretion of IFN- γ secretion of inflammatory cytokines (205).

## Innate immune cells as diagnostic and therapeutic targets

The innate immune system is also involved in immune homeostasis and healthy tissue turnover. This is accomplished *via* three steps consisting of early inflammation, amplification of the inflammatory signal and resolution. Liver fibrosis is a consequence of inflammation and inefficient resolution. Liver biopsy is the gold standard for diagnosing cirrhotic liver disease, yet it is estimated to miss 10-30% of cases (169). Additionally, biopsy is not ideal because of invasiveness, pain, hypertension and bleeding (206). An optimal approach would be to identify early inflammation before fibrosis development. This could improve patient’s treatment and prognosis. Blood markers bring promising perspectives to detect liver damage and abnormal functions (207). The current scoring system for diagnosis and prognosis of fibrosis includes serum proteins (albumin), bilirubin, liver enzymes (aminotransferases, alkaline phosphatase, γ-glutamyl transferase) and direct markers of extracellular matrix turnover (type IV collagen, matrix metalloproteinases). However, there is room for improvement regarding specificity (etiology) and sensitivity (disease stages) (206). The immune system has emerged as an interesting diagnostic and therapeutic target in liver inflammation. Innate immune cells are the frontline defenders in the liver and participate in the initiation, amplification and resolution of inflammation. Identifying immune changes in innate immune cell´s surface expression markers and frequencies can bring future perspective to the diagnosis of low-grade inflammation and also novel therapies. As discussed in this review, depletion of innate immune cells in mice models with hepatitis was able to attenuate several liver diseases. Noteworthy, the close relationship between innate immune cells with DAMPs and cytokines signaling suggests taking into consideration all three factors for the future of liver immunomonitoring and therapies.

## Author contributions

JYZ performed literature search and wrote the manuscript. The author confirms being the sole contributor of this work and has approved it for publication.

## Supplementary material

Supplementary Table 1 Innate immune cells in liver inflammation.
